# Life context of pharmacological academic performance enhancement among university students – a qualitative approach

**DOI:** 10.1186/1472-6939-15-23

**Published:** 2014-03-07

**Authors:** Elisabeth Hildt, Klaus Lieb, Andreas Günter Franke

**Affiliations:** 1 Department of Philosophy, Johannes Gutenberg University of Mainz, Jakob Welder-Weg 18, D – 55099 Mainz, Germany; 2 Department of Psychiatry and Psychotherapy, University Medical Centre, Untere Zahlbacher Str. 8, D – 55131 Mainz, Germany

**Keywords:** Cognitive enhancement, Academic performance enhancement, Stimulants, Ethics, Attitudes, Life impact, University students

## Abstract

**Background:**

Academic performance enhancement or cognitive enhancement (CE) via stimulant drug use has received increasing attention. The question remains, however, whether CE solely represents the use of drugs for achieving better academic or workplace results or whether CE also serves various other purposes. The aim of this study was to put the phenomenon of pharmacological academic performance enhancement via prescription and illicit (psycho-) stimulant use (Amphetamines, Methylphenidate) among university students into a broader context. Specifically, we wanted to further understand students’ experiences, the effects of use on students and other factors, such as pressure to perform in their academic and private lives.

**Methods:**

A sample of 18 healthy university students reporting the non-medical use of prescription and illicit stimulants for academic performance enhancement was interviewed in a face-to-face setting. The leading questions were related to the situations and context in which the students considered the non-medical use of stimulants.

**Results:**

Based on the resultant transcript, two independent raters identified six categories relating to the life context of stimulant use for academic performance enhancement: Context of stimulant use beyond academic performance enhancement, Subjective experience of enhancement, Timing of consumption, Objective academic results, Side effects, Pressure to perform.

**Conclusions:**

The answers reveal that academic performance enhancement through the use of stimulants is not an isolated phenomenon that solely aims at enhancing cognition to achieve better academic results but that the multifaceted life context in which it is embedded is of crucial relevance. The participants not only considered the stimulants advantageous for enhancing academic performance, but also for leading an active life with a suitable balance between studying and time off. The most common reasons given for stimulant use were to maximize time, to increase motivation and to cope with memorizing. According to the interviews, there is a considerable discrepancy between subjective experiences and objective academic results achieved.

## Background

The non-medical use of so-called “smart drugs” among students with the aim of increasing mental performance has attracted considerable media attention over the past few years [1]. In particular, prescription stimulants (methylphenidate, prescription amphetamines e.g. Adderall^®^), other prescription drugs (modafinil, antidementives), as well as illicit drugs (e.g. illicit AMPH, cocaine) have been used in this context [2-4].

Regarding the effects of these “smart drugs”, there is very limited scientific evidence to support the pro-cognitive properties in healthy individuals. Current research data from randomized double-blind placebo-controlled trials (RCTs) among healthy subjects show inconsistent (pro-) cognitive effects on solely simple and higher cognitive domains with stronger pro-cognitive effects on sleep-deprived subjects compared to non-sleep-deprived subjects [5-7]. A recent study revealed the lack of enhancing effects of mixed amphetamine salts on cognitive abilities in healthy young individuals [8]. In spite of this, in anecdotal reports and quantitative and qualitative studies users said to subjectively experience positive effects on cognition [8-10]. In addition, growing evidence indicates that healthy users perceive motivational and emotional effects to be of value in the non-medical use of psychostimulants [11,12].

In the non-medical use of psychostimulants, safety risks and side effects of stimulants (e.g. gastrointestinal symptoms, dizziness, induction of psychosis or mania) as well as misuse, a certain abuse potential and withdrawal symptoms have to be considered [5,13,14]. Beyond that, the distinctions between the use of prescription stimulants and illicit stimulants concerning safety risks, methods of acquisition, legal consequences and costs have to be regarded. In this respect the legal and health risks of the use of illicit stimulants seem to be much higher than of the use of prescription stimulants.

The use of “smart drugs” has been described using various terms including “pharmacological cognitive enhancement” (CE), “(pharmacological) neuroenhancement”, “cosmetic neurology” or “brain doping” [1,4,15]. These terms are usually used to refer to attempts to enhance cognitive functions such as attention, memory or other cognitive domains that are of particular interest for academic performance [16].

Up to now, however, most studies on mental enhancement purposes predominantly focus on the general misuse of prescription and illicit stimulants. In addition to enhancement purposes, these studies also tend to describe other contexts of stimulant misuse, associated with purposes like recreational use, getting high, experimenting, partying, boosting creativity [17,18]. Most recent epidemiological studies of substance use among students increasingly focus on the aspect of “academic performance enhancement” [19-23]. However, the extent to which academic performance enhancement can be separated from partying, getting high and the other above-mentioned purposes remains unclear.

Previous studies revealed a huge range of prevalence rates, results depending on the survey method, type of substance used for CE and surveyed subjects. According to these, the primary purpose and context of the use of drugs (to get high, to increase concentration, recreational use, etc.) seem to be the most important factors amongst the huge range of prevalence rates. Taken together, current research indicates a prevalence rate for these substances for CE of at least 3 – 20% among university students [19-22]. Assuming academic performance enhancement to be a stigmatizing subject, the two most recent studies – which promised a high anonymity to participants – revealed a lifetime prevalence rate of 20% for prescription stimulants (web-based) and a 20% last-year prevalence rate (Randomized Response Technique) for the “use of drugs that you can only buy in a pharmacy for academic performance enhancement” [20,24].

The prevalent use of potential cognitive enhancing drugs on high school and university campuses seems surprising in light of the very limited pro-cognitive effects and sizeable side effects described in literature and given the illegality of misusing prescription and illicit drugs. Several reasons can be assumed for the current interest in so-called pharmacological cognition enhancers in academic contexts [11]. One reason may be recent media hype on the putative cognition-enhancing effects of psychostimulants which may have resulted in the assumption among students that psychostimulants serve to increase academic performance [1,25]. Another reason may be that there is a benefit for at least part of the users, but that up to now this benefit is not adequately reflected in literature. It is also possible that for genetic or other reasons, part of the healthy population is particularly susceptible to cognition-enhancing effects of psychostimulants. Alternatively, there may be a non-cognitive effect of psychostimulant intake that students consider of value in academic contexts [11,12].

In order to find out more about the latter possible explanation and about the real life context of drug use for academic performance enhancement, we ran the present study. In this, leading questions are: Why do students (and other persons) use stimulants in academic contexts? What are the effects users experience and consider of value? Does the use of stimulants in academic contexts provide advantages? How does it impact the students’ lives? What are the side-effects experienced?

Up to now, there is a considerable lack of qualitative research that might be able to answer questions like these. Remarkable exceptions are two studies by DeSantis and colleagues [26,27], Partridge and colleagues [28] as well as the recent interview study by Scott Vrecko, which focuses on the users’ experiences of non-medical stimulant drug use [12]. A recent quantitative study by Ilieva and Farah provides support to the hypothesis that there may be non-cognitive effects of psychostimulant intake that are considered as advantages by healthy individuals [11]. In contrast, the existing epidemiological studies are not able to give explanations for the prevalent phenomenon of academic performance enhancement and can only speculate about deeper reasons and motivational factors. Furthermore, they do not reveal anything about the “every-day” and “real world” effects of stimulant use among students. Currently, in particular the last aspect is a research desideratum.

The present study aims to reduce this lack of empirical scientific data about contextual factors and real world effects of academic performance enhancement. In order to find out more about the reasons for stimulant use, experienced effects of stimulants and their impact on academic results and the user’s life in- and outside university, we conducted semi-structured interviews on a group of university students who were experienced with the use of stimulants for enhancement purposes.

## Methods

### Participants

We posted placards on public bulletin boards throughout the University of Mainz campus asking students who had used prescription or illicit (psycho-) stimulants (amphetamines = AMPH, methylphenidate = MPH, ecstasy, cocaine) for CE purposes to contact us anonymously via email. Placards promised an expense allowance of 30,- Euros for participation. Only healthy students without psychiatric disorders (e.g. attention-deficit hyperactivity disorder (ADHD), schizophrenia) and current physicians’ prescriptions of psychoactive medication (e.g. Ritalin^®^) were included in the study. 22 interviews were carried out.

### Interview guideline

An extensive semi-structured face-to-face interview guideline was developed. After gathering socio-demographic information (age, sex, study subject, grades, etc.) we asked questions concerning the intake of prescription and illicit stimulants with the particular intention of academic performance enhancement and factors associated with this use. Of particular interest here are questions relating to the background of (psycho-) stimulant use, such as “Why did you take the stimulant?”, “Did you experience a considerable increase in mental performance?”, “Did your academic results actually improve as a direct result of taking the stimulant drug?”, “Did you experience negative side effects?”.

### Procedure

A psychologist and three interviewers were trained for the interview procedure. The psychologist examined all participants to ensure that all candidates with psychiatric diseases and current physicians’ prescriptions of psychoactive medication were excluded. Subsequently, each participant was interviewed by two people at a time. The interviews were tape-recorded. To avoid loss of information (e.g. acoustic problems of sound recording), one interviewer asked the questions whilst the other noted down answers to closed questions and catchwords to open questions to complement the tape-recording.

Participants gave written informed consent for being interviewed and for the tape-recording of the entire interview. They received an expense allowance of 30,- Euros when the interview was over. The study was approved by the local Ethics Committee of the Landesärztekammer Rheinland-Pfalz (Medical Association Rheinland-Pfalz).

### Analysis

Records were transcribed verbatim by a person who was not involved in the interview procedure. The anonymous transcriptions were then analyzed systematically using a qualitative approach based on inductive category development [29-31]: Two independent raters (Hildt and Franke) analyzed the transcriptions blindly. The answers given by the participants relating to the impact of the use of prescription and illicit stimulants on individual life context in- and outside university were analyzed and categories subsequently formed. To ensure objective analysis, only those categories upon which both raters agreed were used for the final analysis for this paper.

Based on this method, some preliminary results concerning users’ views on the (moral) differences between caffeine and illicit/prescription stimulants for CE [9] and concerning information transfer [32] have already been published.

## Results

Participant characteristics have already been described in Franke and colleagues (2012) concerning users’ views about (moral) differences between caffeine and prescription/illicit drugs [9]. To summarize, only some (n = 30) students contacted us via telephone or e-mail. Having corresponded with these candidates to identify potential exclusion criteria and following a further planning procedure, 22 interviews were carried out. 4 interviews were not analyzed because of diagnosed ADHD, Pseudologia fantastica or technical reasons. 18 interviews entered further analysis.

Among all participants (n = 18 = 100%), 77.8% (n = 14) had used illicit stimulants (AMPH) and 38.9% (n = 8) prescription stimulants (MPH). 22.2% (n = 4) had used prescription as well as illicit stimulants for academic performance enhancement.

Participants were 25.8 ± 2.88 years old (mean). 66.7% (n = 12) of all participants were male, 33.3% (n = 6) female. Participants had already completed 7.35 ± 3.79 semesters (mean) and were attached to three different departments (Humanities: 44.4%, n = 8; Natural Sciences: 33.3%, n = 6; Economics: 22.2%, n = 4).

**Figure 1:**
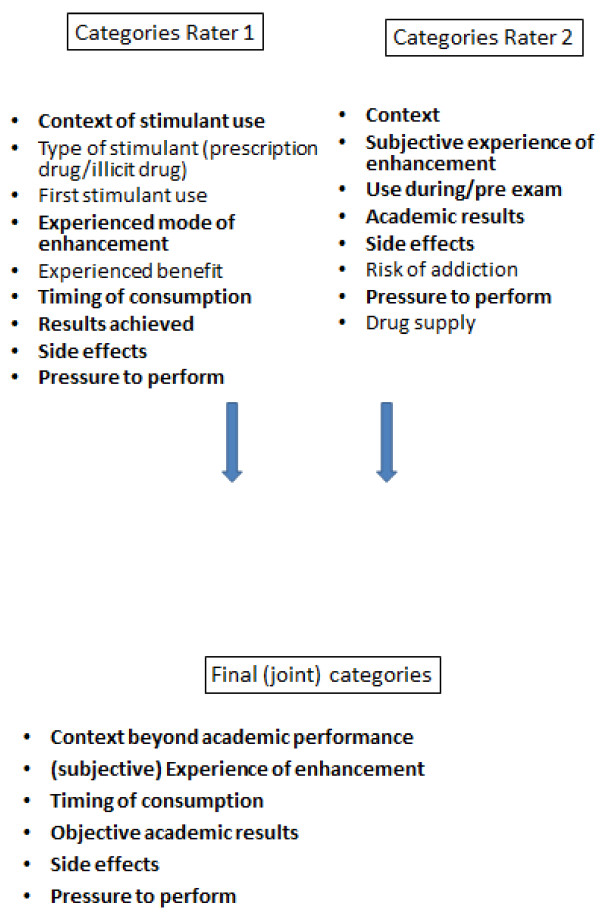
Procedure of building categories for analysis.

The average age of first stimulant use for CE was 20.4 ± 2.88 years. Frequency of stimulant use for academic performance enhancement varied widely from nonrecurring use up to daily use during specific periods of time, e.g. during exam preparation.Based on the selected answers concerning the impact of stimulant use on the students’ personal life, categories were formed independently by two raters. Rater 1 created 9 categories and rater 2 created 8 categories. The raters agreed on 6 categories. Categories with congruent contents that were formed by both raters were used for further analysis (Figure 1). Here we partly reproduce direct speech as stated by the interviewees to give a lively insight into the embedded context of CE.

### Category 1: context of psychostimulant use beyond academic performance enhancement

Several students did not limit their use of stimulants (AMPH) to academic ends, but also resorted to them for other purposes, such as composing music or for a band performance. This indicates that they also used stimulants for non-academic purposes. Some of them said they primarily used illicit AMPH during leisure time and only rarely in order to improve mental performance for academic purposes (“I took it three or four times as a performance enhancer, but even more often just in my free time.”).

Other participants reported that stimulant use helped them to better pursue the projects they value outside of university so that they had time and energy left for leisure activities (“Usually I have more in my head than I have time for. So when I wanted to do other things apart from studying, I took it, too.”). A participant said that it enabled him to begin a career in music and to continue studying at university at the same time. Another student reported that AMPH stimulants served to prolong computer game sessions. Another participant claimed to use AMPH in order to brighten up trivial activities, such as cleaning his flat. One student took MPH to fight fatigue during a night of waiting at the airport.

Several of the students who used illicit AMPH said they first took it for recreational purposes or partying and only later came to know that it might be useful for academic purposes (“Primarily I started taking it as a party drug. I didn’t become aware of the positive effect in other parts (like studying) until later”).

### Category 2: timing of consumption

In academic contexts, most of the students interviewed used stimulants for studying and exam preparation. Some used them in order to write a term paper or to prepare a presentation. Several of them said it allowed them to study for longer periods of time without breaks and to be less prone to boredom (“The other students who didn’t take it (MPH) were able to study for about 3 to 4 hours during the exam phase and afterwards they were shattered and couldn’t do anymore. Sometimes I sat 12 hours in the library and I studied all the way through with hardly any breaks.”).

For others, the main benefit was to gain time or compensate for lost time. These students might take the drug if they left too little time to prepare for an exam (“I thought: ‘Oh no, only 3 days to go till the exam, I don’t know a thing and I need to know it all by heart. What should I do?’ And then I took Ritalin for three days and hardly slept.”).

Some students took stimulants just on the night before the exam in order to prepare for the test. According to them, AMPH is particularly suitable in this respect insofar as it reduces the sleep requirement (“Sometimes when I knew: ‘Alright tomorrow there’s a test and I haven’t done anything again’. Then I took it in the evening and I went to school in the morning without any sleep having spent the night studying.”; “The problem is, if you study the night before, then you have to take amphetamines again the next morning.”).

A considerable number of students used stimulants, particularly prescription stimulants, during exams. However, some students viewed the side effects of (illicit) AMPH as problematic during exams or in situations with some kind of “external control” (“I tend to sweat more and talk faster and I have a faster flow of words. And in my opinion, this is conspicuous.”). Nevertheless, some students were on AMPH during exams.

Whereas most participants used CE predominantly during exam periods, i.e. for 2-3 weeks or sometimes longer, a few of the students took the drugs for longer periods of time: one student because he felt permanently under time pressure, and another reported to have taken (illicit) AMPH regularly for an extended time period in the past as a consequence of its addictive effects.

### Category 3: subjective experience of enhancement

We received multifaceted responses to the question inquiring to what extent the students experienced an actual increase in performance as a result of drug use. A considerable number of students reported an increase in concentration and receptiveness and in their ability to focus on the learning material. The used stimulant was considered particularly to be helpful in situations in which the brain is required to record large amounts of information. A typical answer is: “I could focus better and I wasn’t as distracted.”

Some participants described an increase in their motivation to revise (“…normally, when I have to study for long I get bored. (…) That’s the difference I noticed when I started taking Adderall. I didn’t really want to stop…”). Others stressed the ability to achieve more in a limited space of time as the principal reason for using stimulants (“What you could see as an effect is that I finished quicker which saved me time. I don’t think I got better, though.”).

A student participant emphasized that he experienced an improvement in memory functions (“Usually I have to get things into my short-term memory and then into my long-term memory, basically just reading through a couple of times. Under Ritalin it was enough to read over it quickly and I was able to remember it days afterwards without Ritalin. Well, as I said, the things that I study with Ritalin stay there longer.”).

Another participant reported a positive effect with regard to learning simple content (e.g. vocabulary); however, the beneficial effects were less pronounced when it came to more complex material and negative effects were mentioned when attempting to learn and understand complex material (“If you have to understand the link between everything, it (AMPH) wasn’t a big help at all. So AMPH is good for everything that you just have to learn by heart. AMPH was good in that moment. But when it gets more complicated then AMPH is just obstructive.”).

Others described the effects of (illicit) AMPH as even more ambivalent. A student said: “Reading a text with AMPH is quite nice, you’re just concentrated and awake, but your attention isn’t that much better and because of that your memory is not working that well either. What was that on the other page? It’s there but not well connected. It’s like a virtual picture in your head, you can access it but it’s not connected at all.” One participant reported of lack of concentration and inability to focus on a defined task under illicit amphetamines.

Beyond that, users of (illicit) AMPH also considered other effects to be of relevance in their stimulant use. For some students, an important aspect of AMPH intake is that less sleep is required which leads to an acquisition of time. For instance, AMPH enabled them to learn during the night before their exam (“The advantage is the opportunity to be awake and concentrated which doesn’t really make studying better though. It’s just gaining time.”). Other participants value an increase in creativity (“You have loads of ideas that you never had before.”) Some participants also suggested that AMPH improved communication: “[In the German written exam] I took it because I felt that the flow of words and what you say increases, so writing down what you’re trying to put into words gets easier and even more creative”.

### Category 4: objective academic results

A crucial question with regard to the relevance of CE in academic performance is whether participants’ results actually improved. The answers obtained were inconsistent: although most students considered the use of stimulants to have had some positive influence, only few participants were confident that the drug had a direct positive impact on their grades. Others said that they did not achieve better results in exams or that they were unclear as to whether the drug helped improve their grades. A typical answer regarding AMPH use for academic performance enhancement was: “It was only the feeling I had. There wasn’t really a difference between the results with or without taking it.”

Nevertheless, many of them appreciated some other effect connected to the use of stimulants: that stimulant use increased their motivation, helped them meet deadlines (e.g. for term papers), or increased their learning capacity and effectiveness of learning (“I think I could have gotten the good results also without Ritalin. But because I always leave the things that I don’t like till last minute, I can say that it did help me. But maybe I couldn’t be bothered to study without taking it.”; “For me it’s just a motivation thing.”).

Another student reflected on the correlation between effort and results (“You’re able to study more in less time because of an increased attention. So if I’d spend more time for studying the results would have been the same as with the stuff [AMPH]. When I consider the time and the effort, then the result was better, yes.”).

In the students’ reflections on whether or not the stimulant drugs had beneficial effects, the distinction between the subjective impression of experiencing some kind of increase in mental performance and the objective effects attached to the drug use played a considerable role. It raises the question as to whether the supposed positive effect is simply a subjective feeling or whether it actually has an enhancing effect (“In that moment I felt as if it was better. But the results weren’t as good as I expected, especially with amphetamines”; “At the time, [when under the influence by AMPH] you feel as if you understand it quicker, maybe that it’s of considerable use. But afterwards, when you have another look, maybe study again afterwards or talk about it with somebody then it shows that it was more obstructive than beneficial, at least in my experience.”).

### Category 5: side effects

The students experienced considerable side effects including tachycardia, sleeplessness, restlessness and tremor. Several participants said that in spite of the side effects experienced, they went on taking the stimulant because they appreciated its positive effects.

Side effects observed depended on the duration of intake. With regard to illicit AMPH, a participant said: “After a day [of AMPH use] everything is fine, but after two days it gets worse and on the third day you’re just shattered.”; “It depends on the duration of the intake (AMPH). You do get kind of tired because it is consumptive, so I can’t take it more than once or twice a month. I used to take it every day and it was really crappy because I was completely exhausted.”

Some students claimed to have been struck by depressive moods as a result of MPH use (“It’s some sort of stimulant, you feel high and then it’s like a rollercoaster. Like the rollercoaster going up and then down again. It’s sort of like that. This depressive mood [after the use of stimulant drugs], it’s really not worth it. To me it’s a very unpleasant feeling.”). One of them reported on more long-term depressive feelings from MPH use. He said that he managed to suppress the depressive mood by taking another MPH tablet the following day. In addition, he reported a considerable influence of the psychostimulant on his social life (“I completely separated myself from the outside world while I took Ritalin. In light of this I don’t know whether my depressive mood was because of Ritalin. Or maybe it was just that I hardly had any more contact with friends and that I didn’t go out at a night anymore. Instead I spent the whole day at the university.”).

After a prolonged period of illicit AMPH abuse, a participant reported having developed addictive behaviour.

### Category 6: pressure to perform

Several participants explicitly mentioned pressure to perform as the main reason for stimulant use. One person talked about compensating pressure to perform by using stimulants for recreational purposes in this way: “I also take it to balance the work-related stress and the pressure to perform.” Another participant talked about pressure to perform in academic contexts and about the perceived need to adapt to the educational system and its needs as follows: “The thought of there being so much to do for school and of turning into some sort of a machine really wore me down.” Another student said, “[…] I want to be better than my peer group and fellow students, although I realize that that sounds rather anti-social. But when it comes to group work, it really gets to me when my fellow students contribute so little. […] Personally, I care about getting good marks, particularly in my bachelor thesis, and ending up with a well-paid job. Although it is perhaps selfish, I think it’s o.k. [to use CE substances].”

## Discussion

All students interviewed used prescription stimulants, in particular MPH (Ritalin^®^) or illicit AMPH, for academic performance enhancement. However, there were also other purposes (recreational use, partying, etc.). This is in accordance with a meta-analysis by Wilens and colleagues [18]. This broad spectrum of uses partly explains why, to date, empirical knowledge concerning prevalence rates relating to pure academic enhancement is scarce. In addition, the data reveal that, in particular, those who used illicit AMPH in the beginning often did so in the context of partying and only later switched to (also) using the substances for academic performance enhancement. This suggests that stimulant use may spread from recreational use and partying to academic contexts.

Several participants reported having taken stimulants not so much to improve academic results but to achieve an adequate work-life-balance and to have time and energy left for leisure activities. According to the results obtained, some users consumed stimulants in order to pursue their individual projects outside the academic sphere. This aspect sheds new light on CE, which is often perceived solely as a strategy to exceed natural cognitive capacity in competitive situations at school, university or in the workplace. Instead, if one takes the users’ overall life situation into consideration, it seems that they perceive stimulants at least partly as beneficial for leading an “active life” without being focused too much on academics. The results obtained here are well in line with a recent publication by Partridge and colleagues, who tentatively suggest that one motivation for stimulant use among students may be to maintain an active social life [28].

When stimulants were used for academic purposes, the main reasons listed were to facilitate exam preparation, to gain study time and to help prepare a term paper. Time pressure and pressure to perform are of considerable relevance here. Stimulants were also taken on exam days to improve performance. From this it is plausible to assume that during exam periods or during other periods of high academic stress, stimulant use among university students increases, this assumption coincides with recent results obtained by wastewater analysis of amphetamine and ritalinic acid at a US college campus [33].

Interestingly, when asked whether stimulant intake was helpful for obtaining better academic results, the participants gave rather ambivalent answers. According to the interviewees, it is not clear whether stimulant intake had objective positive effects on academic performance or, contrarily, whether it was of no help at all or even obstructive.

This inspires the question: What are the benefits of psychostimulant use? To what extent does it increase the mental performance or cognitive abilities of healthy individuals?

The answers we received indicate that the benefits experienced do not include improved cognitive abilities such as planning or complex information processing but rather help with learning by heart. The results seem to imply that psychostimulant intake has a positive motivational effect which fosters concentration, receptiveness and ability to focus. This effect has been confirmed by previous studies [4,12]. Furthermore, several students appreciated other beneficial effects such as a reduced need for sleep, increased motivation or the feeling of being “re-energized”. Several students involved in the music scene cited increased creativity as a reason for AMPH use. When we compare these interview results with scientific literature on cognitive enhancers, it seems that users most appreciate the wake-promoting and pro-vigilant effects described in previous studies. According to current scientific data, although wake-promoting substances may promote alertness and concentration at night for learning purposes, they enhance neither memory, nor other higher cognitive domains, nor mood. Furthermore, these very mild pro-cognitive effects have proved more effective in sleep-deprived subjects compared to normal healthy subjects [4].

As underlined by the users’ answers, it is not so much the hope of improving higher cognitive functions which stimulates them to take drugs for academic performance enhancement but, rather, the wake-promoting and motivational effects associated with them.

Taken together, among the main motivations for non-medical use of stimulants by students seem to be the motivation to get things done, be it in order to meet academic demands or to have a fulfilling leisure time, and the motivation to feel active and energetic while performing their tasks. This supports the findings by Partridge and colleagues [28].

Thus, even if the stimulants currently used for enhancement purposes are not very effective as *cognitive* enhancers, i.e. fail to increase complex cognitive functions, they nevertheless seem to be considered advantageous by a certain group of students. It would therefore be inadequate to declare current so-called cognitive enhancers to be useless based on the grounds that they do not effectively increase complex cognitive abilities. Instead, the interviews revealed that the predominant reason why students turn to these drugs today is their motivational and wake-promoting effects.

The main motive explaining why some students use psychostimulants for CE is the prospect of studying in a more efficient and fulfilling way. In this context, maximizing time and boosting motivation are two of the most important aspects. In addition, for the users, one of the most attractive functions of stimulants such as MPH or AMPH is the procurement of a good feeling during exam preparation and exams. This helps them to cope with stressful situations – an effect that could be explained by euphoric effects of psychostimulants.

The results obtained here are in considerable accordance with the study by Vrecko [12] who reported not so much an influence of psychostimulants on cognition but on emotional or affective states. In this qualitative study, based on semi-structured interviews with 24 students who used stimulants for CE, the author identified emotion-related statements in the interviews which he grouped into four categories: “Feeling Up”, “Drivenness”, “Interestedness”, “Enjoyment”. Even if in the study here we did not focus on emotional or affective states, there obviously are several parallels, in particular with regard to the relevance the interviewees conferred to the subjective experience of psychostimulant use. Strikingly, both studies indicate that, for the users, the main motive for stimulant use is to be able to handle the demands of university, in particular, with regard to the practical, motivational and emotional aspects of studying and preparing for exams. This is in perfect line with a recent study by Ilieva and Farah [11] who found out that students who used psychostimulants for enhancement perceived motivational advantages of stimulant use to be at least as pronounced as cognitive effects.

Interestingly, several participants believed that if they had started earlier with exam preparation and taken their studies more seriously, they would have had the same academic results. Stimulants seem to be of particular value to those students who lack motivation or who start preparing for exams at the last minute. However, it is important to stress that there are surely other, non-pharmacological means of achieving these ends. These include reducing time pressure, working in a more disciplined way or improving the balance between university and private life.

Although it is generally presumed in the academic debate that those who avail themselves of so-called cognitive enhancers benefit from drug use, only two of the students interviewed were confident that with stimulant use they achieved better academic results, i.e. better grades. According to the interviews, students who took stimulants (MPH/AMPH) rarely obtained better results during exams. What students seemed to appreciate most about the drug was the increased motivation to study and the time they gained.

The results lead us to question a direct distorting effect of psychostimulants on the grades obtained in university exams, i.e. an effect that benefits the users. On the one hand, at the moment, it seems that students who do not take psychostimulants need not feel disadvantaged or pressured to use enhancers themselves in order to avoid being left behind. On the other hand, however, it may be argued that there may be an indirect advantage to those who use enhancers in terms of motivation or higher learning effectiveness. Furthermore, psychostimulants may confer a certain advantage with regard to time pressure.

In addition, as can be inferred from several answers, there is a difference between people saying that they experienced a positive, enhancing effect from psychostimulant use and the objective, empirically detectable existence of a significant enhancing effect. This concurs with the idea that psychostimulant use is associated with the overestimation of one’s own capabilities. The results obtained here are in accordance with a recent quantitative study on the effects of mixed amphetamine salts on healthy young adults [8]. Overall, the study did not reveal significant enhancement effects on cognitive performance in a set of 13 measures of cognitive ability examined. Nevertheless, compared to those who received a placebo pill, those who had taken a stimulant pill believed their cognitive performance to be more enhanced. It is important to keep the distinction between objective effects and subjective perception of effects in mind as it may help us to avoid an over-optimistic picture of the purported positive, cognition-enhancing effects of stimulants.

The interviews suggest that side effects are considerable, which is in contrast to the results obtained by deSantis and Hane [26]. Side-effects are one important reason why, in most cases, stimulants were not used for a long period of time, but intermittently, interwoven with long periods of abstinence. Several users mentioned sleep deprivation and related consequences, and reported on the need to adjust drug intake in order to avoid seriously disrupting their sleep patterns. When stimulants were taken over a longer period of time (weeks etc.), side effects became aggravated. In particular, some students reported depressive periods once the drug had lost its stimulating effects. Furthermore, one participant stated a development of addictive behaviour. Aspects related to addiction and the users’ views on the risk of addiction will be discussed in more detail in a separate manuscript.

It is important to note that this study is hampered by several limitations. One of them is the limited number of interviews: Only 18 interviews were taken into consideration. In spite of the fact that the University of Mainz has 36.000 registered students who had the possibility to notice the advertising placards of this interview study throughout the campus, only 30 students contacted us, and only 22 were willing to participate. Given CE prevalence rates of 3 – 20%, there should have been a much higher number of potential participants for this study. We hypothesize that the stigmatizing subject of this study is the reason for the low participation rate, notwithstanding the fact that anonymity was guaranteed and that participants were remunerated for their time and effort with 30,- Euros.

The “type” of student willing to participate may display the main bias of the small group of participants. Participants had to be frequently enough at the campus, had to have enough time for the interview study, had to be interested in participating in scientific studies, had to be convinced about the importance of the CE phenomenon, had to take the risk to talk about their illegal behaviour without being punished, etc. This kind of a “self-selection” of participants may have led to a certain bias in the composition of the group of participants.

In addition, the exclusion of students with psychiatric disorders and current physicians’ prescriptions of psychoactive medication leads to bias implying that the present study is not representative for the entirety of students. The prevalence rate of psychiatric disorders among the population is high – e.g. 5.3% for ADHD among children/adolescents, 4.4% among adults [34,35]. All these students have been excluded which displays an important bias. However, it was not the aim of this study to explore CE aspects among patients but among healthy subjects. Including ADHD patients would have meant to include participants with a totally different context of stimulant use (misuse of own prescription medication) and would have diluted the distinction between therapy of disorders and enhancement of healthy people’s abilities. It is questionable whether we excluded potential participants who faked or exaggerated ADHD symptoms for a prescription of stimulants by a physician.

Furthermore, the interviewees gave us their spontaneous answers to the questions we asked in the interviews. The answers we obtained are in no way exhaustive or comprehensive, nor can we assume that the participants frankly told us everything that came to their minds. Instead, the answers represent an extract that may be influenced by aspects such as present time frame, social desirability or the participants’ perceived need to justify their behaviour. Nevertheless, it is reasonable to assume that, overall, the answers represent the actual views and experiences of the interviewees since the interviews were held in a very relaxed atmosphere and the participants obviously did not refrain from stating drawbacks and doubts concerning CE.

The data obtained in this preliminary qualitative study is in no way representative. It is important to stress that based on the interviews, we are not able to draw definite conclusions on the effects of stimulant intake for enhancement purposes in healthy individuals in general. The answers represent the purely subjective views of the interviewees. Nevertheless, the reports are very valuable because they give us insights on the factors which lead students to use stimulants for academic performance enhancement.

## Conclusions

The results obtained reveal that academic performance enhancement through the use of stimulants is not an isolated phenomenon that solely aims at enhancing cognition to achieve better academic results but that the multifaceted life context in which it is embedded is of crucial relevance. The participants not only considered the stimulants advantageous for enhancing academic performance, but also for leading an active life with a suitable balance between studying and time off. The most common reasons given for stimulant use were to maximize time, to increase motivation and to cope with memorizing. According to the interviews, there is a considerable discrepancy between subjective experiences and objective academic results achieved.

More research needs to be carried out to better understand the practice and context of academic performance enhancement. The results obtained here may serve as a starting point for future qualitative and quantitative research. It will be necessary to know more about the students’ motivations for pharmacological neuroenhancement and about its objective effects on academic performance achieved. In addition, research should be done on drug use put in the context of other strategies for coping with pressure to perform in academic contexts which will include questions such as: What other ways are there to cope with pressure that do not involve the misuse of drugs? To what extent does society play a role in the field of neuroenhancement? All of this will form the basis for future policy recommendations concerning pharmacological neuroenhancement.

## Abbreviations

ADHD: Attention deficit hyperactivity disorder; AMPH: Amphetamine(s); CE: Cognitive enhancement; MPH: Methylphenidate; RCT(s): Randomized double-blind placebo-controlled trial(s).

## Competing interests

All authors declare that they have no competing interest.

## Authors’ contributions

EH, AGF and KL participated in the conception and design of the study. Interviews were performed by EH, AGF, CB and MC. DL did the transcription of the interviews. AGF and EH have analyzed the data and have built categories. All authors participated in data interpretation, drafting, and revising the manuscript. All authors read and approved the final manuscript.

## Authors’ information

AGF and KL belong to the Department of Psychiatry and Psychotherapy, University Medical Centre Mainz, Germany. KL is head of the Department of Psychiatry and Psychotherapy; AGF is a trainee of this department. EH belongs to the Department of Philosophy and is an expert in Neuroethics.

## Pre-publication history

The pre-publication history for this paper can be accessed here:

http://www.biomedcentral.com/1472-6939/15/23/prepub
